# Metabolic improvement and liver regeneration by inhibiting CXXC5 function for non-alcoholic steatohepatitis treatment

**DOI:** 10.1038/s12276-022-00851-8

**Published:** 2022-09-16

**Authors:** Seol Hwa Seo, Eunhwan Kim, Minguen Yoon, Soung-Hoon Lee, Byung-Hyun Park, Kang-Yell Choi

**Affiliations:** 1 Department of Biotechnology, College of Life Science and Biotechnology, Yonsei University, Seoul, 03722 Republic of Korea; 2 CK Regeon Inc, Seoul, 03722 Republic of Korea; 3 Department of Biochemistry and Molecular Biology, Chonbuk National University Medical School, Jeonbuck, 54896 Republic of Korea

**Keywords:** Metabolic syndrome, Metabolic syndrome, Regenerative medicine

## Abstract

Non-alcoholic steatohepatitis (NASH) is a chronic liver disease that results from multiple metabolic disorders. Considering the complexity of the pathogenesis, the identification of a factor mediating the multiple pathogenic phenotypes of NASH will be important for treatment. In this study, we found that CXXC5, a negative feedback regulator of the Wnt/β-catenin pathway, was overexpressed with suppression of Wnt/β-catenin signaling and its target genes involved in hepatic metabolism in obese-NASH patients. *Cxxc5*^*−/−*^ mice were found to be resistant to NASH pathogenesis with metabolic improvements. KY19334, a small molecule that activates the Wnt/β-catenin pathway via interference of the CXXC5-Dvl interaction, reversed the overall pathogenic features of NASH as *Cxxc5*^*−/−*^ mice. The improvement in NASH by KY19334 is attributed to its regenerative effects through restorative activation of the suppressed Wnt/β-catenin signaling. Overall, the pronounced metabolic improvements with the stimulation of liver regeneration by interfering with the CXXC5-Dvl interaction provide a therapeutic approach for NASH.

## Non-alcoholic liver disease: Treatment to stop suppression of crucial signaling pathway

A small molecule treatment that restores the suppression of important signaling pathways could be effective against obesity-related liver disease. Non-alcoholic steatohepatitis (NASH) is a severe and prevalent condition in obese and diabetic patients. No specific NASH medications exist, but studies have shown that the Wnt/β-catenin signaling pathway is crucial in activating key genes that fight the disease. Kang-Yell Choi at Yonsei University in Seoul, South Korea, and co-workers investigated the role of CXXC5, which suppresses the Wnt/β-catenin pathway. They showed that mice with *Cxxc5* knocked out were resistant to the worst effects of NASH. The researchers developed a treatment using a small molecule called KY19334 that interferes the function of CXXC5 via inhibition of its binding with Disheveled. This treatment suppressed inflammation, steatosis, oxidative stress and cell death, reversed liver fibrosis and enhanced liver regeneration.

## Introduction

Non-alcoholic fatty liver disease (NAFLD) is prevalent among obese and diabetic patients^1,2^. Insulin resistance plays a critical role in the pathological process of NAFLD by increasing the free fatty acid (FFA) flux to the liver, resulting in hepatic steatosis and lipotoxicity^3^. Non-alcoholic steatohepatitis (NASH) is the most severe form of NAFLD and is often caused by hepatic steatosis, chronic inflammation, and liver injury-associated fibrosis^2^. Although NASH is a severe and prevalent liver disease, a clinically applicable medication for its treatment is not available^4,5^. Due to the complexity of the developmental process and the multiple pathological phenotypes, including insulin resistance, steatosis, inflammation, and fibrosis^6,7^, the development of drugs that cure one or several disease phenotypes of NASH has limited treatment efficacy^8–10^. Therefore, understanding the overall pathogenic process of NASH and identifying a factor that drives the multiple pathological phenotypes are attractive for the development of effective treatments for NASH patients.

The Wnt/β-catenin signaling pathway plays key roles in a wide range of metabolic disorders, such as inflammation, hepatic steatosis, gluconeogenesis, energy metabolism, and insulin resistance, which may be attributed to its target genes that play critical roles in metabolism, including glucagon-like peptide-1 (*GLP-1*), transcription factor-7-like-2 (*TCF7L2*), peroxisome proliferator-activated receptor delta (*PPARδ*), and WNT1-inducible signaling pathway protein 1 (*WISP1*)^10^. Wnt/β-catenin signaling is suppressed in the liver tissue of NASH patients, and the expression of its target genes involved in multiple pathological phenotypes of NASH is subsequently suppressed. For example, GLP-1, a direct transcriptional target of Wnt/β-catenin signaling, and its agonizts are suppressed by increased body weight in obese diabetic patients^11^. GLP-1 agonizts, such as liraglutide, exenatide, and semaglutide, are known to reduce hepatic steatosis in NAFLD patients by directly affecting lipid metabolism^12^. The inhibitor of dipeptidyl-peptidase 4 (DPP4), a GLP-1 destabilizer that is transcriptionally induced by Wnt/β-catenin signaling, prevents multiple NASH phenotypes^13,14^. In addition, activation of the Wnt/β-catenin pathway stimulates the neogenic replacement of damaged tissues such as the liver^15^, and overexpression of β-catenin after hepatotoxic liver injury enhances its regeneration via activation of its target genes^16,17^. Leucine-rich repeat-containing G-protein coupled receptor 5 (*LGR5*), one of the major Wnt/β-catenin signaling target genes involved in the activation of adult stem cells, plays a role in the self-renewal of tissues such as the small intestine, colon^18^, stomach^19^, hair follicles^20^, and liver^21^.

CXXC5-type zinc finger protein 5 (CXXC5) has been identified as a negative feedback regulator of the Wnt/β-catenin signaling pathway that functions through a direct interaction with the disheveled (Dvl) protein^22,23^.

In this study, we found that CXXC5 was specifically induced with suppressing Wnt/β-catenin pathway target genes such as *GLP1*, *TCF7L2*, *WISP1*, and fos-related antigen 1 (*FOSL1*) in the liver tissues of NASH patients with metabolic disorders. Multiple pathological phenotypes of NASH, including inflammation, steatosis, oxidative stress responses, and fibrosis, were not significantly observed in *Cxxc5*^*−/−*^ mice. CXXC5 is specifically induced in the cytosol of cells in the adipose and liver tissues of NASH patients. CXXC5, especially cytosolic CXXC5, which binds Dvl, as a target for the development of drugs to treat NASH was confirmed by the similar improvement of NASH phenotypes in *Cxxc5*^*−/−*^ mice and with oral application of KY19334, a small molecule interfering with CXXC5-Dvl protein‒protein interaction (PPI)^24^. The improvement in the multiple pathological phenotypes of *Cxxc5*^*−/−*^ mice and with KY19334 treatment could be achieved by restoration of Wnt/β-catenin signaling followed by the activation of multiple target genes involved in NASH pathogenesis and liver tissue regeneration. Overall, a small molecule approach abolishing the cytosolic function of CXXC5 via interference of CXXC5-Dvl PPI is a potential treatment for improving NASH.

## Materials and methods

### Animal studies

The generation of *Cxxc5*^*−/−*^ mice has been previously described^23^. *Cxxc5* heterozygous mice were intercrossed for four generations to obtain littermate wild-type and *Cxxc5*^*−/−*^ mice and maintained on a C57BL/6 background. *Cxxc5*^*+/+*^ and *Cxxc5*^*−/−*^ mice exhibited NASH following HFD feeding containing CCl_4_ (Sigma‒Aldrich) or gold thioglucose (GTG, Sigma‒Aldrich). For the CCl_4_-induced NASH mouse model, 6-week-old male mice (KOATECH, Korea) were fed a 60% kcal HFD (Research Diet, D12492) for 12 weeks and administered 0.05 ml/kg CCl_4_ twice a week, and for the final 4 weeks, they were daily administered KY19334 (25 mg/kg), selonsertib (25 mg/kg, Selleckchem), ocaliva (25 mg/kg, ApexBio Technology) or vehicle^25^. For the GTG-induced NASH mouse model, 5-week-old male mice were intraperitoneally injected with GTG, followed by NCD for 1 week. Then, the mice were fed a HFD (Orient Yeast, F2HFD2) or NCD for the next 11 weeks. The HFD-fed mice were orally administered KY19334 (25 mg/kg), selonsertib (25 mg/kg), ocaliva (25 mg/kg) or vehicle^25^ for the final 4 weeks. For the glucose tolerance test (GTT) or insulin tolerance test (ITT), mice were injected with D-glucose (1.5 g/kg body weight, Sigma-Aldrich) after overnight fasting or human insulin (0.75 U/kg body weight, Gibco) after 4 h of fasting. Tail blood was drawn at 0-, 15-, 30-, 60-, 120-, and 180-min intervals, and blood glucose levels were measured using a One Touch Ultra glucometer (LifeScan). All animals were housed on a 12-h light/12-h dark cycle, with free access to food and water. All protocols were reviewed and approved by the Institutional Review Board of Severance Hospital, Yonsei University College of Medicine (09-013).

### Blood chemistry

The total blood of the mice was collected by cardiac puncture after fasting. The blood was allowed to clot for 30 min and then centrifuged for 10 min at 1000 × *g* to obtain the supernatant. The supernatant was used to measure the metabolic parameters. ELISA kits were used to assess serum insulin (Milliporem Burlington, MA, USA), serum FFAs (Cayman Chemical), and serum adiponectin (ABclonal). The homeostasis model assessment of insulin resistance (HOMA-IR) was used to calculate insulin resistance. Serum parameters, including total cholesterol, high-density lipoprotein (HDL) cholesterol, triglycerides (TGs), alanine aminotransferase (ALT), and aspartate aminotransferase (AST) concentrations, were measured using a blood chemistry analyzer (FUJI DRI-CHEM 4000i). Calibration was performed using the quality control card supplied with the FUJI DRI-CHEM slides whenever slides from a new lot were used.

### Cytokine profile

Cytokines and chemokines in mouse serum were assessed using the Mouse XL Cytokine Array Kit (Proteome Profiler Mouse XL Cytokine Array; R&D system), arrowing simultaneous detection of the expression levels of 111 different liver damage-related proteins. The array was performed according to the manufacturer’s instructions. Blots were developed with enhanced chemiluminescence using a LAS-3000 luminescent imaging analyzer (Fujifilm).

### H&E staining

The dissected tissues were fixed in 4% neutral paraformaldehyde (PFA) and embedded in paraffin. The paraffin sections were cut to a thickness of 4 μm and subjected to H&E staining. Adipocyte cell size was measured in 20 randomly chosen microscopic areas from three independent animals using a Nikon bright-field optical microscope (Nikon TE-2000U). The average adipocyte size was determined using ImageJ software.

### Immunohistochemistry (IHC)

Paraffin sections of human liver tissue microarray (Xenotech) and mouse liver and scWAT were deparaffinized and rehydrated. For antigen retrieval, slides were autoclaved in 10 mM sodium citrate buffer (pH 6.0). To block endogenous peroxidase activity, tissues were incubated with 0.345% H_2_O_2_ (Samchum Chemicals) for 30 min. Before incubating the sections with mouse primary antibody, mouse IgG was blocked using a M.O.M Mouse IgG blocking kit (Vector Laboratories). Sections were incubated with the following primary antibodies overnight at 4 °C: anti-β-catenin (BD, 610154, 1:100), anti-CXXC5 (Lab made, 1:100), anti-LGR5 (Abnova, PAB2591, 1:10), anti-CD133 (eBioscience, 14-1331-82, 1:100), anti-HNF4a (Santa Cruz Biotechnology, sc-374229, 1:50), and anti-Ki67 (Abcam. ab15580, 1:100). The slides were washed with PBS, incubated with Alexa Fluor 488- (Invitrogen, A-11001) or Alexa Fluor 555-conjugated IgG secondary antibody (Invitrogen, A-21428) at 20 °C for 1 h, and counterstained with DAPI (Sigma-Aldrich, D9564). The images were captured using an LSM700 META confocal microscope (Carl Zeiss) after excitation with 405-, 488-, or 543-nm lasers. To block endogenous peroxidase activity before peroxidase IHC analysis, tissues were incubated with 0.345% H_2_O_2_ (Samchun Chemicals) for 30 min. Before incubating the sections with mouse primary antibody, mouse IgG was blocked using a M.O.M Mouse IgG blocking kit (Vector Laboratories). Sections were incubated with primary antibodies overnight at 4 °C at the following dilutions: anti-PPARγ (Santa Cruz Biotechnology, sc-271392, 1:100), anti-F4/80 (Santa Cruz Biotechnology, sc-377009, 1:100), anti-CD11b (Abcam, 133357, 1:100), anti-α-SMA (Abcam, ab7817, 1:50), anti-collagen I (Abcam, ab34710, 1:50), anti-desmin (Abcam, ab15200, 1:100), and anti-UCP1 (Abcam, ab10983). Sections were then incubated with biotinylated anti-rabbit (Dako, BA-1000) secondary antibodies for 1 h at 20 °C. The samples were stained with 3,3-diaminobenzidine (DAB; Dako, SK-4100) for 3–7 min and counterstained with Mayer’s hematoxylin (Muto). All incubations were conducted in a humid chamber. Signals were analyzed using a bright-field microscope (Nikon TE-2000U).

### Isolation of liver macrophages and progenitor cells for FACS analysis

Immediately after sacrifice, the liver tissues were excised and washed with PBS to remove blood. Tissues were minced and digested with 1 mg/mL collagenase type IV (Sigma‒Aldrich) in PBS containing 150 U/mL DNase I for 30 min at 37 °C. The digested liver was suspended in 50 mL of DMEM, and the dissociated cells were filtered through a 100-μm nylon strainer and collected by centrifugation at 300 × *g* for 4 min. Stromal vascular fraction pellets were resuspended in 0.5 ml of RBC lysis buffer (BioLegend) for 5 min on ice, centrifuged for 5 min at 300 × *g*, and resuspended in PBS. Cells were incubated with the following fluorescently labeled antibodies for 30 min: anti-F4/80-FITC (eBioscience, 11-4801-82, 1:10) or anti-CD11b-PE (eBioscience, 12-0112-82).

Isolation of LPCs was performed as previously described^21,26^. Red cells were lysed with buffer (160 mM NH_4_Cl, KHCO_3_ containing 0.01% EDTA) and resuspended in Williams E medium (Gibco) with 10% FCS. Following centrifugation at 300 × *g* for 5 min at 4 °C, the supernatant was collected, washed twice, and resuspended in PBS with 2% FCS for FACS staining. LPCs were stained with anti-LGR5 (Abnova, PAB2591, 1:10) at 4 °C for 30 min and then incubated with Alexa 555-conjugated IgG secondary antibody (Invitrogen, A-21428) at 4 °C for 10 min. The stained cells were analyzed by flow cytometry (BD Bioscience).

### Sirius red collagen staining

Paraffin-embedded liver tissue sections were deparaffinized and rehydrated. Tissues were stained with Weigert’s solution for 8 min and subsequently with picrosirius red for 1 h.

### DHE staining

Cryo-liver tissue sections were counterstained with DAPI (Sigma-Aldrich) and washed with PBS. The sections were then incubated with dihydroethidium (DHE; 1:10,000) for 30 min at 37 °C in the dark and washed with PBS. The samples were mounted in 100% glycerol, and reactive oxygen species (ROS) levels in the liver were observed using an LSM700 META confocal microscope (Carl Zeiss) after excitation with a 405-nm laser.

### Oil Red O (ORO) staining

Cryo-liver tissue sections were fixed in PFA for 15 min and washed with 70% isopropanol (Duksan Pure Chemicals). The lipid droplets were stained with ORO solution (Sigma-Aldrich) at 20 °C overnight. The samples were counterstained with Mayer’s hematoxylin. Images of ORO staining were visualized using a bright-field microscope.

### TG assay

Liver tissues were incubated on ice in 100 μL of saline solution (2 M NaCl, 2 mM EDTA, 50 mM sodium phosphate, pH 7.4). Tissue suspensions were assayed for TG content using a triglyceride assay kit (Cayman Chemical).

### RNA extraction and quantitative PCR

Total RNA was extracted from ground tissue powder using TRIzol reagent (Invitrogen) according to the manufacturer’s instructions. Reverse transcription was performed using M-MLV reverse transcriptase (Invitrogen) and 2 μg of total RNA. The synthesized cDNA was diluted to a concentration of 100 ng/μL, and quantitative PCR analyses were performed in the Rotor-gene Q real-time PCR cycler (Qiagen) using SYBR green reagent (Qiagen) under the following conditions: 95 °C for 10 min followed by 40 cycles at 95 °C for 5 s and 60 °C for 15 s. Relative amounts of mRNA were quantified using the comparative Ct method (∆∆Ct). All mRNA values were normalized to those of *GAPDH*. The primer sequences are listed in Supplementary Table 3.

### Bioinformatics data analysis

Molecular pathway dysregulation in human visceral adipose tissues was determined by gene set enrichment analysis, surveying the molecular pathway gene set in the Molecular Signature Database (MsigDB) (www.broadinstitute.org/msigdb). Cross-species comparison of transcriptomic dysregulation was performed in the space of molecular pathway gene sets from the HALLMARK and KEGG databases, and statistically significant dysregulation was defined as a false discovery rate (FDR) < 0.01 in either of the two human liver tissue transcriptome datasets: normal (*n* = 12) vs. NASH (*n* = 13) subjects (GSE48452).

### Statistical analyses

Information about statistical details and the methods is indicated in the figure legends, tests, or methods. Data are presented as the mean ± SD. Statistical analyses were performed using an unpaired two-tailed Student’s *t test*. Asterisks denote statistically significant differences (*P < 0.05; **P < 0.01; ***P < 0.001).

## Results

### CXXC5 is induced in liver tissues of obesity-related NASH

**Fig. 1:**
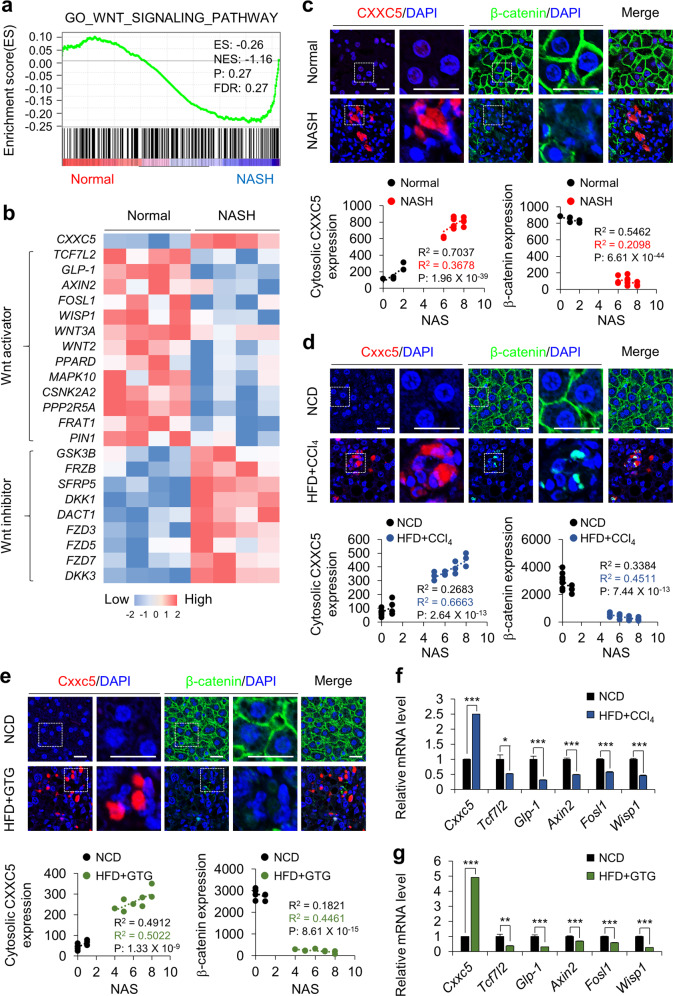
The Wnt/β-catenin pathway inhibitor CXXC5 was induced in the liver tissues of patients and mice with NASH. **a** Gene set enrichment analysis of microarray transcriptome data for normal and NASH patients for the Wnt/β-catenin signaling-activated gene signature. Black columns indicate 83 enriched genes involved in the Wnt/β-catenin signaling pathway in the liver tissue of obese patients with all stages of NASH and controls (NASH, *n* = 18; control, *n* = 14). NES normalized enrichment score, ES enrichment score, FDR false discovery rate. **b** Hierarchical clustering and heatmap of RNA-seq data in liver tissues from morbidly obese patients with NASH (Normal, *n* = 4; NASH, *n* = 4). The color scale shows Z score fragments per kilobase of transcript per million mapped reads representing the mRNA levels of each gene in the blue (low expression) to red (high expression) color scheme. **c**–**e** Representative images of CXXC5 and β-catenin expression in human liver (**c**), HFD + CCl_4_− (**d**), and HFD + GTG-induced NASH mouse (**e**) tissues. Graphs in the bottom panels represent quantitative analyses of IHC staining for correlation of the expression of cytosolic CXXC5 and β-catenin with the NAS using covariance in the liver tissues from normal and NASH human subjects (Normal, *n* = 5; NASH, *n* = 12), those from NCD and HFD + CCl_4_-induced mice (NCD, *n* = 12; HFD + CCl_4_, *n* = 12), and those from NCD and HFD + GTG-induced mice (NCD, *n* = 8; HFD + GTG, *n* = 8). Scale bars, 100 µm. **f**, **g** Relative mRNA expression of *Cxxc5* and Wnt/β-catenin signaling target genes (*Tcf7l2*, *Glp-1*, *Axin2*, *Fosl1*, and *Wisp1*) in the livers of NCD- and HFD + CCl_4_-induced NASH mice (NCD, *n* = 12; HFD + CCl_4_, *n* = 12) (**f**) and NCD- and HFD + GTG-induced NASH mice (NCD, *n* = 8; HFD + GTG, *n* = 8) (**g**). Data represent the mean ± SD. *P < 0.05; **P < 0.01; ***P < 0.001 determined by Student’s *t* test.

To elucidate the clinical implications of the role of CXXC5 in NASH development, we investigated the mRNA expression profiles of CXXC5 and Wnt-responsive genes in NASH patients with obesity (Supplementary Table 1) using gene set enrichment analysis (Gene Expression Omnibus (GEO): GSE48452). The results of the microarray analyses showed that the mRNA levels of the Wnt/β-catenin signaling target genes involved in metabolism, such as *TCF7L2*, *GLP1*, *AXIN2*, *FOSL1*, and *WISP1*, were lower in liver tissues from NASH patients (Fig. 1a, b). However, the mRNA and protein levels of CXXC5, a negative regulator of Wnt/β-catenin signaling, were higher in the liver tissues of NASH patients than in those of normal subjects (Fig. 1b, c and Supplementary Table 2). In contrast, β-catenin expression was suppressed in the liver tissues of NASH patients, although that in normal subjects was highly expressed (Fig. 1c).

To confirm the role of CXXC5 in animal models, we used HFD plus carbon tetrachloride (HFD + CCl_4_)- or HFD plus gold thioglucose (HFD + GTG)-induced NASH model mice (Supplementary Figs. 1, 2). The protein and mRNA levels of Cxxc5 were highly increased in the liver tissues of mice with NASH induced by HFD + CCl_4_ or HFD + GTG (Fig. 1d–g). On the other hand, β-catenin was expressed in hepatocytes of NCD-fed mice but was decreased in cells from NASH-induced mice (Fig. 1d, e). Moreover, quantitative analyses confirmed that CXXC5 and β-catenin levels were correlated and inversely correlated, respectively, with the NAFLD activity score (NAS) in liver tissues of both patients with NASH and NASH-induced mice (Fig. 1c–e; bottom panel). The expression of Wnt/β-catenin signaling response genes, such as *Tcf7l2*, *Glp-1*, *Axin2*, *Fosl1*, and *Wisp1*, which are involved in metabolic dysfunction, was lower in the liver tissues of HFD + CCl_4_− or HFD + GTG-induced NASH mice (Fig. 1f, g). These results indicate that CXXC5 may be a key factor in NASH development.

### Cxxc5 induces metabolic dysfunction

**Fig. 2:**
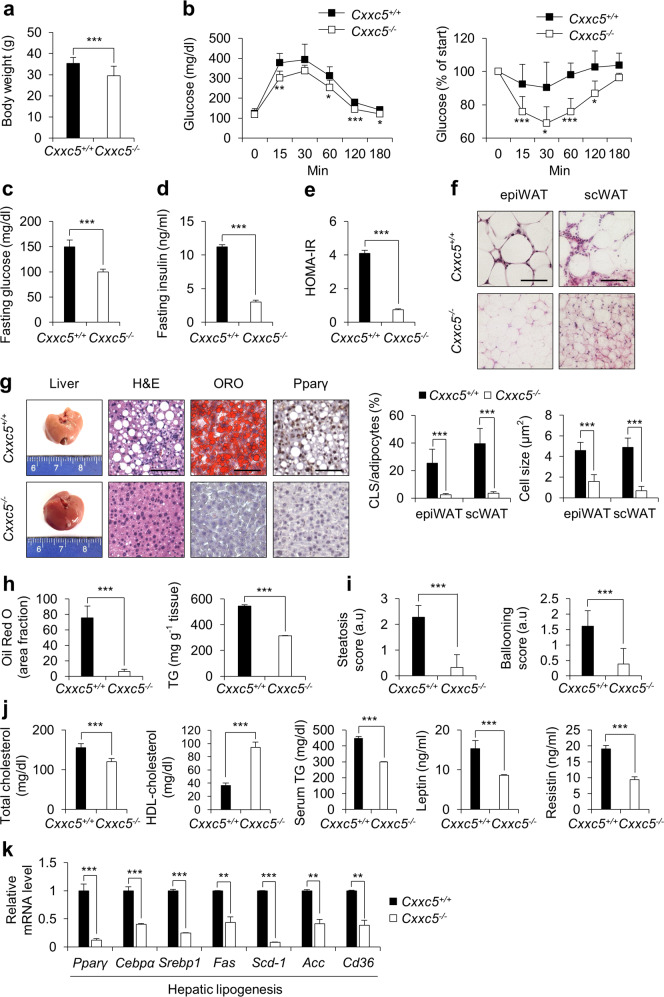
Deletion of *Cxxc5* ameliorates hepatic steatosis with metabolic improvement. **a**–**k** *Cxxc5*^*+/+*^ and *Cxxc5*^*−/−*^ mice were fed a HFD for 12 weeks and injected with CCl_4_ twice a week for the final 4 weeks to induce NASH (*n* = 7 per group). **a** Body weight. **b** GTT and ITT. **c** Fasting glucose. **d** Plasma insulin concentration in the overnight fasted state. **e** HOMA-IR. **f** Representative images of H&E staining of epiWAT and scWAT (upper panel). Quantification of CLSs and cell size in adipocytes (lower panel). Scale bars, 100 µm. **g** Representative histological analyses of images of liver tissue; H&E staining, ORO staining, and Pparγ staining. Scale bars, 100 µm. **h** Ratio of the ORO-positive area (left panel) and TG concentration (right panel) in the liver. **i** The scores of steatosis (left panel) and ballooning (right panel). **j** Serum concentrations of total cholesterol, HDL-cholesterol, TGs, leptin, and resistin. **k** Relative mRNA expression of genes involved in hepatic lipogenesis (*n* = 3). Data represent the mean ± SD. *P < 0.05; **P < 0.01; ***P < 0.001 determined by Student’s *t* test.

To confirm the role of CXXC5 in the pathogenesis of NASH, *Cxxc5*^*+/+*^ and *Cxxc5*^*−/−*^ mice were fed HFD + CCl_4_. The *Cxxc5*^*−/−*^ mice exhibited lower body weight than the *Cxxc5*^*+/+*^ mice and significantly improved systemic glucose tolerance and insulin sensitivity (Fig. 2a–d), as confirmed by HOMA-IR analyses (Fig. 2e). The lower body weight of *Cxxc5*^*−/−*^ mice correlated with a reduction in adipogenic phenotypes in adipose and liver tissues (Fig. 2f–h). The number of crown-like structures (CLSs) and the size of adipocytes were decreased in both epididymal white adipose tissue (epiWAT) and subcutaneous white adipose tissue (scWAT) of the *Cxxc5*^*−/−*^ mice (Fig. 2f). Liver tissues from *Cxxc5*^*−/−*^ mice did not accumulate lipid droplets with the decrease in TG content (Fig. 2g, h). In addition, nuclear expression of Pparγ, the master regulator of adipogenesis^27^, was critically suppressed in liver tissue cells of *Cxxc5*^*−/−*^ mice (Fig. 2g). A reduction in hepatosteatosis was observed in parallel with lower steatosis and ballooning scores in *Cxxc5*^*−/−*^ mice (Fig. 2i). Moreover, the serum metabolic parameters, including total cholesterol, HDL-cholesterol, TGs, leptin, and resistin, were improved in *Cxxc5*^*−/−*^ mice (Fig. 2j). Finally, the expression levels of hepatic lipogenic genes were significantly lower in *Cxxc5*^*−/−*^ mice (Fig. 2k). Overall, Cxxc5 mediates metabolic dysfunction and hepatosteatosis induced by HFD + CCl_4_, as shown by the resistance of *Cxxc5*^*−/−*^ mice.

### Ablation of Cxxc5 resists the development of NASH

**Fig. 3:**
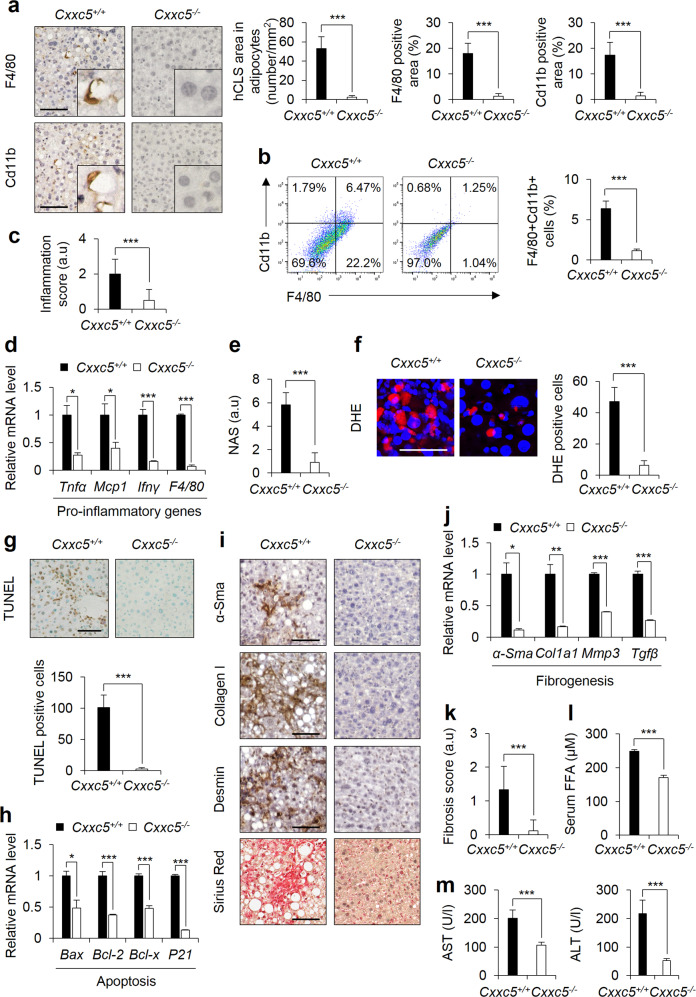
Deletion of *Cxxc5* ameliorates NASH progression. **a**–**m** *Cxxc5*^*+/+*^ and *Cxxc5*^*−/−*^ mice were fed a HFD for 12 weeks and injected with CCl_4_ twice a week for the final 4 weeks to induce NASH (*n* = 7 per group). **a** Representative IHC images of F4/80- and Cd11b-stained liver tissues (left panel). Quantification of hCLS formation and F4/80- and Cd11b-positive areas (right panel). Scale bars, 100 µm. **b** Flow cytometry analyses of the expression of F4/80 and Cd11b (left panel). Percentage of F4/80^+^Cd11b^+^ cells (*n* = 3) (right panel). **c** Inflammation score. **d** Relative mRNA expression levels of genes involved in pro-inflammation (*n* = 3). **e** The NAFLD score. **f** Representative images of DHE-stained cells (left panel). Quantification of ROS formation by measuring DHE-positive cells in liver tissues (right panel). Scale bar, 100 µm. **g** TUNEL staining of the liver (top panel). Quantification of TUNEL-positive cells (bottom panel). Scale bar, 100 µm. **h** Relative mRNA expression of genes involved in cell death pathways (n = 3). **i** Representative IHC images for α-Sma, collagen I, desmin, and Sirius red staining of liver tissues. Scale bars, 100 µm. **j** Relative mRNA expression of genes involved in fibrogenesis (*n* = 3). **k** The fibrosis score. **l**, **m** Plasma concentrations of FFAs (**l**), AST and ALT (**m**). Data represent the mean ± SD. *P < 0.05; **P < 0.01; ***P < 0.001 determined by Student’s *t* test.

The administration of CCl_4_ combined with HFD induces chronic inflammation and oxidative stress, followed by liver fibrosis as in NASH^28,29^. The numbers of F4/80- and Cd11b-positive macrophages were apparently lower in the liver tissue of *Cxxc5*^*−/−*^ mice than in the tissue of *Cxxc5*^*+/+*^ mice with induced NASH (Fig. 3a, b). These results were associated with a reduction in the inflammation score and with the expression of M1 macrophage markers in *Cxxc5*^*−/−*^ mice (Fig. 3c, d). The NAS implied that other NASH phenotypes, including steatosis, ballooning, and inflammation, were lowered in *Cxxc5*^*−/−*^ mice (Fig. 3e). Additionally, hepatic reactive oxygen species (ROS) production was reduced in *Cxxc5*^*−/−*^ mice (Fig. 3f). Hepatic cell death was also significantly reduced in *Cxxc5*^*−/−*^ mice, as shown by the terminal deoxynucleotidyl transferase dUTP nick end labeling (TUNEL) assay and the expression of genes involved in apoptosis (Fig. 3g, h). Liver fibrosis was mostly not observed, as shown by the histological analyses with reduced mRNA levels of fibrogenic markers, including alpha smooth muscle actin (α-Sma), collagen type 1 alpha 1 (Col1a1), metalloprotein-3 (Mmp-3), and transforming growth factor beta (Tgfβ) (Fig. 3i–k). Reduced liver damage in *Cxxc5*^*−/−*^ mice was confirmed by serum FFA, AST, and ALT levels (Fig. 3l, m). Therefore, targeting CXXC5 is a potential approach for the development of agents to treat NASH.

### KY19334 improves obesity and metabolic dysfunctions in mouse NASH models

**Fig. 4:**
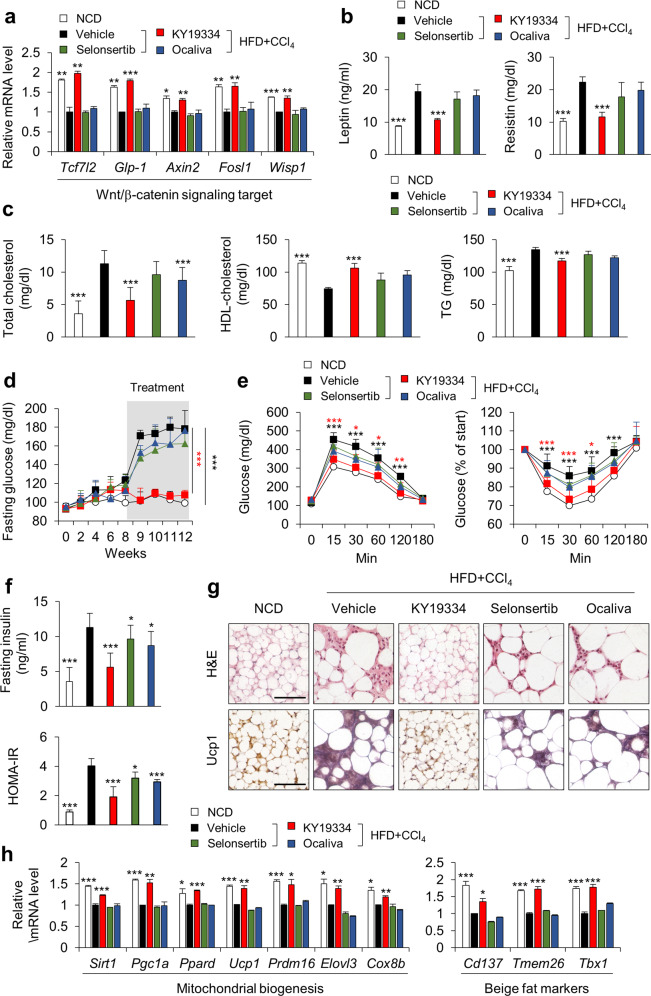
KY19334 treatment improves glucose metabolism and insulin sensitivity in NASH mice. **a**–**h** C57BL/6 mice fed a NCD or HFD + CCl_4_ were orally administered KY19334, selonsertib, or ocaliva at 25 mg/kg/d for 4 weeks (*n* = 10 per group). **a** Relative mRNA expression of Wnt/β-catenin signaling target genes (*Tcf7l2*, *Glp-1*, *Axin2*, *Fosl1*, and *Wisp1*) (*n* = 3). **b** Serum concentrations of leptin and resistin. **c** Serum concentration of total cholesterol, HDL-cholesterol, and TGs. **d** Fasting glucose. **e** GTT and ITT. **f** Plasma insulin concentration in the overnight fasted state (top panel) and HOMA-IR (bottom panel). **g** Representative images of H&E and IHC staining for Ucp1. Scale bars, 100 µm. **h** Relative mRNA expression of genes that are markers of mitochondria and beige fat (*n* = 3). Data represent the mean ± SD. *P < 0.05; **P < 0.01; ***P < 0.001 determined by Student’s *t* test.

As an approach to abolish CXXC5 function and determine its role in the pathological phenotypes of NASH, we examined the effects of KY19334, a small molecule that activates the Wnt/β-catenin pathway by inhibiting its interaction with Dvl^24^. This approach allows the specific blockade of the function of cytosolically overexpressed CXXC5 in order to activate suppressed Wnt/β-catenin signaling target genes involved in cellular metabolism (Fig. 4a). The roles of KY19334 in the suppression of systemic metabolic dysfunction in the progression of NASH were investigated by its oral administration, and its effect was compared with the effects of selonsertib and ocaliva at an identical concentration (25 mg/kg of body weight) or vehicle (Supplementary Fig. 3a). KY19334-treated mice showed improved serum metabolic parameters, including total cholesterol, HDL-cholesterol, TGs, leptin, and resistin, compared to those of selonsertib- or ocaliva-treated mice (Fig. 4b, c). As assessed by protein chip analyses, KY19334 reduced the protein expression of several metabolic, proinflammatory, and fibrogenic cytokines and chemokines, linking the induction of the overall phenotype of NASH (Supplementary Fig. 3b). Glucose tolerance and insulin sensitivity were most significantly improved by KY19334 administration (Fig. 4d–f). Notably, KY19334 promoted browning of scWAT, as shown by the specific induction of mitochondrial biogenesis markers, including thermogenin uncoupling protein 1 (UCP1) and beige-fat markers, which were reduced in HFD + CCl_4_-induced NASH in mice (Fig. 4g, h). However, the mRNA levels of both mitochondrial biogenesis and beige-fat markers did not change in the scWAT of mice administered selonsertib or ocaliva (Fig. 4h). Overall, blockade of the Wnt/β-catenin signaling inhibitory function of CXXC5 improves systemic metabolic abnormalities.

### KY19334 attenuates hepatic steatosis in NASH mice with restorative activation of the suppressed Wnt/β-catenin pathway

**Fig. 5:**
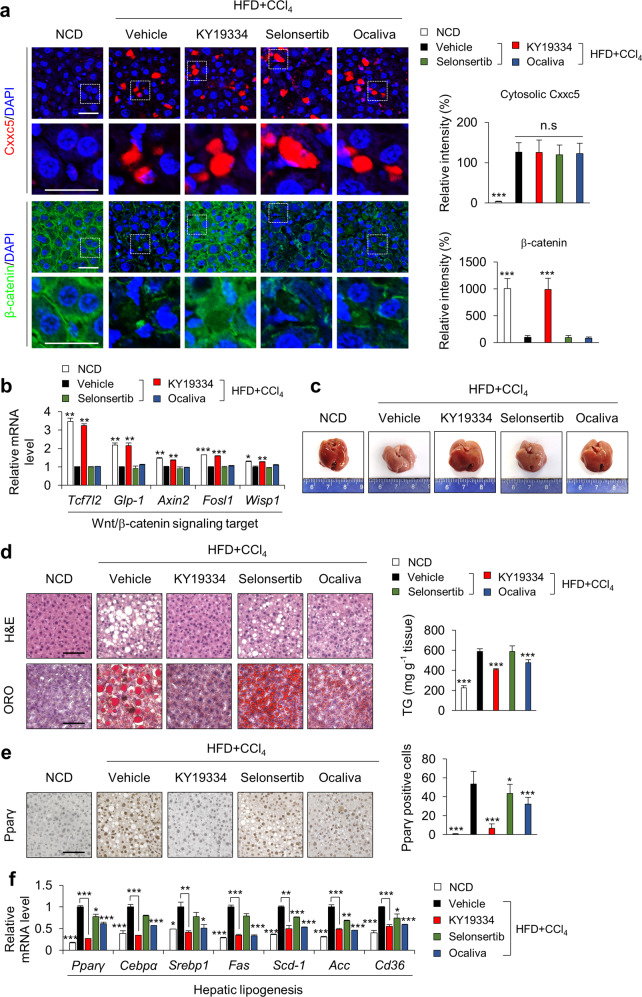
KY19334 activates the Wnt/β-catenin pathway and suppresses steatohepatitis in NASH-induced mice. **a**–**f** C57BL/6 mice fed a NCD or HFD + CCl_4_ were orally administered KY19334, selonsertib, or ocaliva at 25 mg/kg/d for 4 weeks (*n* = 10 per group). The liver tissues were analyzed by various histochemical and biochemical methodologies. **a** Representative images of Cxxc5 and β-catenin (left panel) and quantitative analyses of IHC staining for cytosolic Cxxc5 and β-catenin (right panel). **b** Relative mRNA expression of Wnt/β-catenin signaling target genes (*Tcf7l2*, *Glp-1*, *Axin2*, *Fosl1*, and *Wisp1*) (*n* = 3). **c** Representative gross images of the liver. **d** Representative histological images of H&E staining and ORO staining (left panel). Quantification of the TG concentration in the liver tissue (right panel). Scale bars, 100 µm. **e** Representative IHC images of Pparγ-stained liver tissue (left panel). Quantification of Pparγ-positive cells (right panel). Scale bar, 100 µm. **f** Relative mRNA expression of genes involved in hepatic lipogenesis (*n* = 3). Data represent the mean ± SD. *P < 0.05; **P < 0.01; ***P < 0.001 determined by Student’s *t* test.

The therapeutic effectiveness of KY19334 in NASH was evaluated by examining its effects on the pathological phenotypes of the liver. Cytosolic Cxxc5 was highly expressed in the liver tissue cells of HFD + CCl_4_-induced NASH mice (Fig. 5a). However, compared to the vehicle-, selonsertib-, and ocaliva-treated mice, elevation of β-catenin following activation of Wnt/β-catenin target genes was observed in the liver tissue cells of KY19334-treated mice (Fig. 5a, b). In support of a correlation between β-catenin and steatosis, the NASH-induced mouse livers were a milky pale color indicating fatty liver, which was not significantly observed in mice administered KY19334 (Fig. 5c). The histological features of liver tissues showed critical suppression of steatosis by KY19334, and its effect was much more prominent than that of ocaliva or selonsertib, which showed no significant effect on the reduction of hepatic steatosis as evaluated by ORO staining and measurement of the levels of TGs (Fig. 5d). In addition, KY19334-treated mice showed a more significant reduction in nuclear accumulation of Pparγ than mice treated with selonsertib or ocaliva (Fig. 5e). Similarly, the mRNA expression levels of the hepatic lipogenic genes were more significantly suppressed by KY19334 (Fig. 5f), confirming the reduction in hepatic steatosis by KY19334 treatment.

### KY19334 suppresses hepatic inflammation, oxidative stress, and apoptosis associated with NASH

**Fig. 6:**
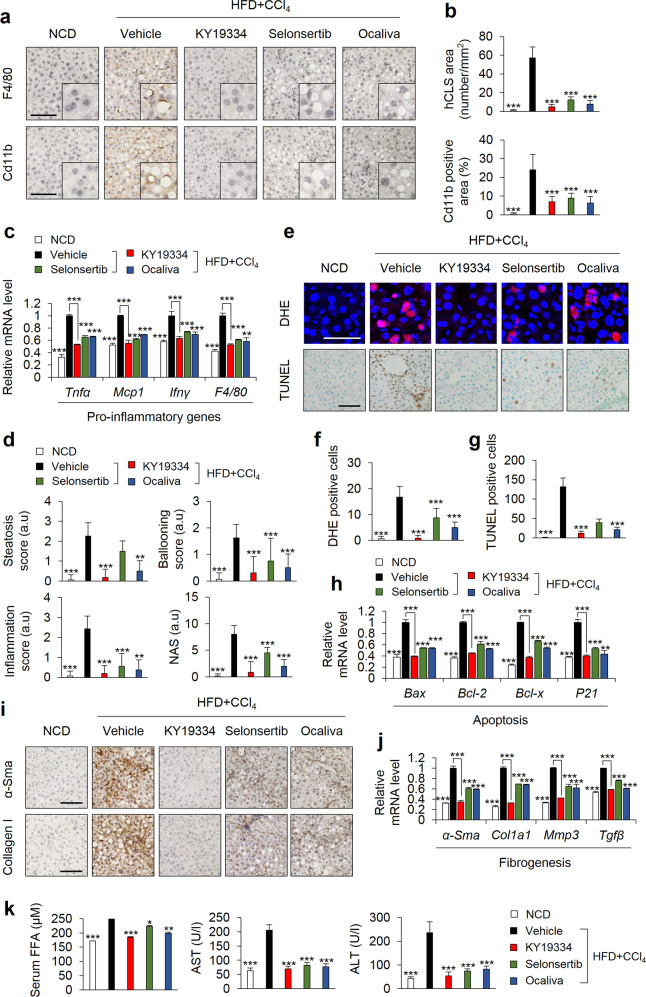
KY19334 suppresses hepatic inflammation and fibrosis in NASH-induced mice. **a**–**k** C57BL/6 mice fed a NCD or HFD + CCl_4_ were orally administered KY19334, selonsertib, or ocaliva at 25 mg/kg/d for 4 weeks (*n* = 10 per group). The liver tissues were analyzed by various histochemical and biochemical methodologies; **a** Representative IHC images of F4/80- and Cb11b-stained liver tissue. Captures are high magnification of hCLSs. Scale bars, 100 µm. **b** Quantification of hCLS formation (top panel) and the Cd11b-positive area (bottom panel). **c** Relative mRNA expression of genes involved in pro-inflammation (*n* = 3). **d** The scores of steatosis, ballooning, inflammation, and NAFLD. **e** Representative images of DHE- or TUNEL-stained livers. Scale bar, 100 µm. **f** Quantification of ROS formation by measuring DHE-positive cells. **g** Quantification of TUNEL-positive cells. **h** Relative mRNA expression of genes involved in cell death pathways (*n* = 3). **i** Representative IHC images of α-Sma and collagen I staining. Scale bars, 100 µm. **j** Relative mRNA expression of genes involved in fibrogenesis (*n* = 3). **k** Serum concentration of FFAs, AST, and ALT. Data represent the mean ± SD. *P < 0.05; **P < 0.01; ***P < 0.001 determined by Student’s *t* test.

As shown by monitoring F4/80^+^ and Cd11b^+^ cells and crown-like structures in the liver tissue, the inflammation induced by HFD + CCl_4_ was suppressed by treatment with KY19334, selonsertib, or ocaliva (Fig. 6a, b). The upregulated hepatic mRNA levels of monocyte/macrophage infiltration markers were all attenuated by KY19334, selonsertib, or ocaliva treatment (Fig. 6c). Although the inflammation induced by HFD + CCl_4_ was similarly suppressed by KY19334, as with selonsertib or ocaliva treatment, the NAS was much more effectively reduced by KY19334 than by selonsertib or ocaliva (Fig. 6d). Oxidative stress was induced in the liver tissues of the HFD + CCl_4_-induced NASH mice, and this was mostly suppressed by KY19334, although it was only weakly reduced by selonsertib or ocaliva (Fig. 6e, f). Consistently, as monitored by the TUNEL assay, cell death was abolished by KY19334, but selonsertib or ocaliva showed a weak effect (Fig. 6e, g). The expression levels of apoptotic markers were markedly reduced in the liver tissue of KY19334-treated mice (Fig. 6h). Collectively, KY19334 suppresses oxidative stress, apoptosis, and hepatic inflammation during NASH development.

### KY19334 reverses liver fibrosis associated with NASH

We next evaluated the effects of KY19334 on hepatic fibrosis as a consequence of chronic liver injury using HFD + CCl_4_-model mice. Liver fibrosis monitored by the accumulation of α-Sma, collagen I, desmin, and Sirius red staining, as well as by measuring the mRNA levels of α-Sma, Col1a1, Mmp-3, and Tgfβ1, was significantly decreased by KY19334 treatment compared to treatment with selonsertib or ocaliva (Fig. 6i, j and Supplementary Fig. 4a). The suppression of fibrosis by KY19334 was much more significant than that observed after treatment with selonsertib or ocaliva (Supplementary Fig. 4b). In addition, NASH-induced increases in serum parameters indicating liver damage, including FFAs, AST, and ALT, were more significantly inhibited by KY19334 treatment than by selonsertib or ocaliva treatment (Fig. 6k). Together, these results demonstrate that KY19334 effectively reverses the progression of liver fibrosis during NASH development.

### KY19334 enhances liver regeneration in NASH mice

**Fig. 7:**
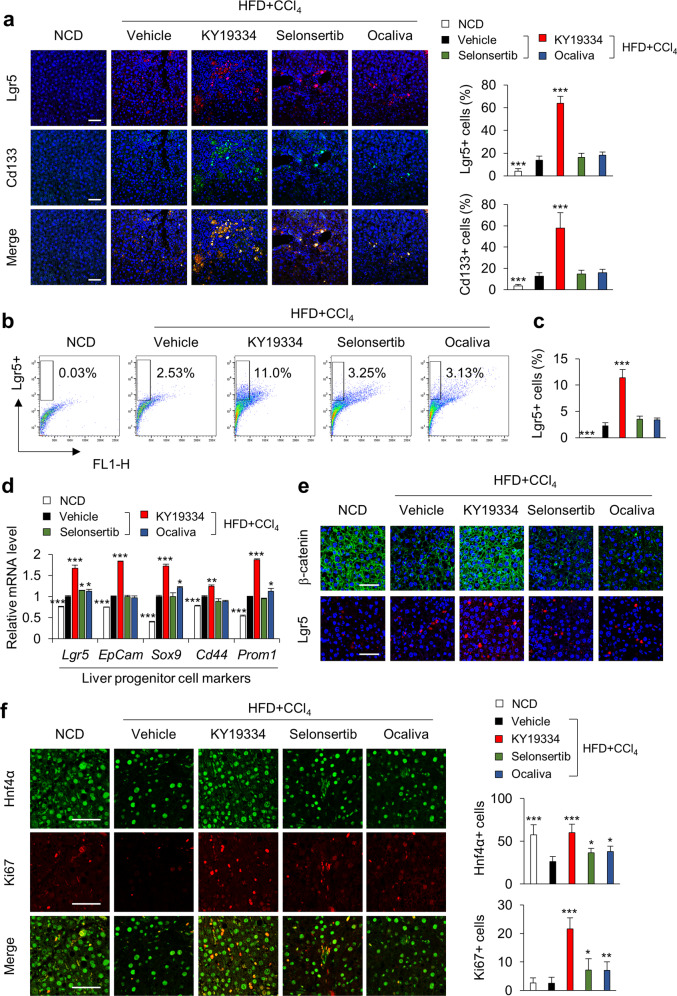
KY19334 promotes regeneration of liver tissue in NASH-induced mice. **a**–**f** C57BL/6 mice fed a NCD or HFD + CCl_4_ were orally administered KY19334, selonsertib, or ocaliva at 25 mg/kg/d for 4 weeks (*n* = 10 per group). The liver tissues were analyzed by various histochemical and biochemical methodologies. **a** Representative IHC images stained for Lgr5 or Cd133 (left panel). Quantification of Lgr5- and Cd133-positive cells (right panel). Scale bars, 100 µm. **b** Flow cytometry analyses of the expression of Lgr5 (*n* = 3). **c** Quantification of Lgr5-positive cells by flow cytometry analyses. **d** Relative mRNA expression of Lgr5 and liver progenitor cell markers (*n* = 3). **e** Representative IHC images stained for β-catenin or Lgr5. Scale bars, 100 µm. **f** Representative IHC images stained for Hnf4α or Ki67 (left panel). Quantification of Hnf4α+ and Ki67+ cells (right panel). Scale bars, 100 µm. Data represent the mean ± SD. *P < 0.05; **P < 0.01; ***P < 0.001 determined by Student’s *t* test.

The prominent effects of KY19334 on the reversion of multiple NASH phenotypes led us to investigate its role in the activation of stem cells residing in the liver. Lgr5, the transcriptional target of the Wnt/β-catenin pathway involved in the activation of stem cells and subsequent tissue regeneration^21^, and the Cd133 stem cell marker were induced by liver damage in HFD + CCl_4_-induced NASH mice (Fig. 7a–c). The numbers of Lgr5+ and Cd133+ cells were significantly increased by administration of KY19334 but not by selonsertib or ocaliva (Fig. 7a–c). The regenerative effect of KY19334 was further confirmed by the overlapping staining of cells for Lgr5+ and Cd133+ (Fig. 7a). The mRNA expression of hepatic progenitor markers, including EpCam, Sox9, Cd44, and Prom1, was also induced by KY19334 but not by selonsertib or ocaliva in the liver tissues of NASH-induced mice (Fig. 7d). The increase in Lgr5+ cells was correlated with β-catenin expression following KY19334 treatment (Fig. 7e). Moreover, KY19334 promoted the proliferation of hepatocytes, as shown by the increase in Ki67 and the hepatocyte progenitor marker Hnf4α (Fig. 7f). The liver regenerative effect of KY19334 is attributed to the control of cytosolic CXXC5 function, as confirmed by the similar increases in Lgr5+ cells in *Cxxc5*^*−/−*^ mice with NASH induced by an identical model system (Supplementary Fig. 5a–f). Overall, the recovery of liver fibrosis by blocking the inhibitory function of Cxxc5 on Wnt/β-catenin signaling correlates with activation of the regenerative system in the liver tissue of NASH mice.

## Discussion

NASH is one of the most prevalent chronic liver diseases associated with metabolic abnormalities, leading to cirrhosis and hepatocellular carcinoma (HCC)^8,30^. Therefore, the treatment of patients at the stage of NASH is essential to prevent the progression to cirrhosis and subsequent HCC. However, currently, a clinically applicable drug for the treatment of NASH is not available, which may be due to the complex pathophysiological features of NASH and the absence of a strategy to restore the injured liver to normal tissue^31–33^.

In the present study, we suggest a new approach for improving NASH phenotypes by restoring the suppressed regenerative Wnt/β-catenin signaling by inhibiting CXXC5 function through either *Cxxc5* knockout or CXXC5-Dvl PPI inhibition. This therapeutic approach is highly effective due to the restoration of the suppressed Wnt/β-catenin signaling target genes involved in liver regeneration and cellular metabolism. The pathological significance of controlling CXXC5 function was rationalized by the overexpression of CXXC5 following suppression of Wnt/β-catenin signaling and subsequent induction of its target genes in the liver tissue of NASH patients.

Various direct Wnt/β-catenin signaling target genes, including *GLP1*, *PPARδ*, *TCF7L2*, *AXIN*, and *WISP1*, and indirect effectors, such as *PPARγ* and *C/EBPα*, which are suppressed by WISP1, are involved in metabolic diseases and have been used as targets for the development of drugs^34^. For example, a GLP1 receptor agonist has been used for the development of clinically available drugs for diabetes and has also been tested as a therapeutic agent for NASH^35,36^. Induction of PPARδ expression, a direct transcriptional target of Wnt/β-catenin signaling^37^, by oral administration of KY19334 implies that systemic energy expenditure was increased by activation of this pathway. Furthermore, ectopic expression of UCP1 in scWAT from KY19334-treated mice was linked to protecting against diet-induced obesity by increasing fatty acid oxidation of scWAT.

The hypothesis that the CXXC5-Dvl PPI is a target for the development of drugs for the treatment of NASH was supported by the improvement in metabolic dysfunctions and pathological features of NASH with the restoration of the suppressed Wnt/β-catenin signaling target genes following KY19334 treatment. In the NASH model induced by HFD combined with CCl_4_, administration of KY19334 increased energy metabolism, accompanied by the enhancement of adipose tissue browning. The role of CXXC5 in the regulation of Wnt/β-catenin signaling was shown by an increase in nuclear β-catenin with the accumulation of cytosolic Cxxc5 in adipocytes of NASH mice. Compared to the ASK-1 inhibitor selonsertib^38^ and the FXR agonist ocaliva^39^, KY19334 showed higher effectiveness in improving the pathological features of the liver in HFD + CCl_4_-induced NASH mouse models when administered at identical concentrations. Steatosis, which was weakly improved by selonsertib, was mostly inhibited by administration of the same amount of KY19334, as shown by various histochemical and biological analyses. Inflammation and ROS-mediated stress and apoptosis were also significantly improved by KY19334 but not by selonsertib or ocaliva.

Finally, fibrosis was not observed in the liver tissue of mice treated with KY19334 compared to mice administered selonsertib or ocaliva, which showed relatively weak improvement, especially in the HFD + CCl_4_-induced NASH model. Similar improvements in NASH phenotypes were also observed in *Cxxc5*^*−/−*^ mice, supporting the therapeutic effects of interference with Cxxc5-Dvl PPI. Moreover, the absence of pathological phenotypes in *Cxxc5*^*−/−*^ mice following NASH induction by either HFD + CCl_4_ or HFD + GTG shows that CXXC5 is a key regulator in the pathogenesis of NASH. The critical role of CXXC5 as a mediator of the pathogenesis of NASH was further confirmed by the absence of CXXC5 induction and any apparent pathological phenotypes by administration of KY19334 when the mice were fed a NCD (Supplementary Fig. 6a–i).

The improvement in multiple NASH phenotypes, including steatosis, ballooning, inflammation, oxidative stress, and fibrosis, by *Cxxc5* knockout or KY19334 treatment could be attributed to the enhancement of the regeneration system through activation of Wnt/β-catenin signaling and subsequent induction of genes involved in regeneration and metabolism, such as *Glp-1*, *Pparδ*, and *Lgr5*^21,40,41^. Lgr5^+^ stem cells involved in liver regeneration accompanied by hepatocyte proliferation^42^ were observed in the liver tissue of NASH mice. The liver tissues of the *Cxxc5*^*−/−*^ mice, as well as those of mice administered KY19334, showed an enhanced liver regeneration system in the HFD + CCl_4_-induced NASH model as monitored by the measurement of the stem cell markers including *EpCam*, *Sox9*, *Cd44*, *Prom1*, and *Lgr5*. The induction of these multiple stem cell markers may be attributed to the activation of direct Wnt/β-catenin signaling target genes, including *Lgr5*^21,26^. The enhancement of liver regeneration by KY19334 in NASH mice was further confirmed by the increased numbers of both Hnf4α- and Ki67-positive cells. Overall, these results indicate that the improvement in the multiple pathological phenotypes of NASH by KY19334 is attributed to the enhancement of the regeneration system of the liver through the restorative activation of the suppressed Wnt/β-catenin pathway.

The enhancement of the regeneration system of the liver by KY19334 provides a further advantage in the efficient activation of the Wnt/β-catenin pathway by its effect on GSK3β inhibition as well as the blockade of CXXC5-Dvl PPI^24^. However, the inhibitory role of KY19334 on GSK3β alone may be limited due to attenuation of Wnt/β-catenin signaling activation by the increase in the negative feedback regulator CXXC5. Therefore, the inhibitory effect of the CXXC5-Dvl PPI is greater than that of GSK3β on the activation of Wnt/β-catenin signaling and the subsequent therapeutic effects of KY19334 on NASH.

Taken together, a small molecular approach, which is able to restore the overall NASH phenotype by metabolic improvement and regenerative tissue restoration, provides a potential therapy for curing chronic fibrotic diseases.

## Supplementary information

Supplemental information
