# Supplementary material for: Metabolic improvement and liver regeneration by inhibiting CXXC5 function for non-alcoholic steatohepatitis treatment
Source: Exp Mol Med. 2022 Sep 16;54(9):1511–23. doi: 10.1038/s12276-022-00851-8 (PMC9534855; doi:10.1038/s12276-022-00851-8)
Supplement: Supplementary file 1 — Supplemental information [file 12276_2022_851_MOESM1_ESM.pdf]

Metabolic improvement and liver regeneration by inhibiting CXXC5 function for non-alcoholic steatohepatitis treatment

Seol Hwa Seo, Eunhwan Kim, Minguen Yoon, Soung-Hoon Lee, Byung-Hyun Park, Kang-Yell Choi

SUPPLEMENTARY INFORMATION

Supplementary Fig. 1

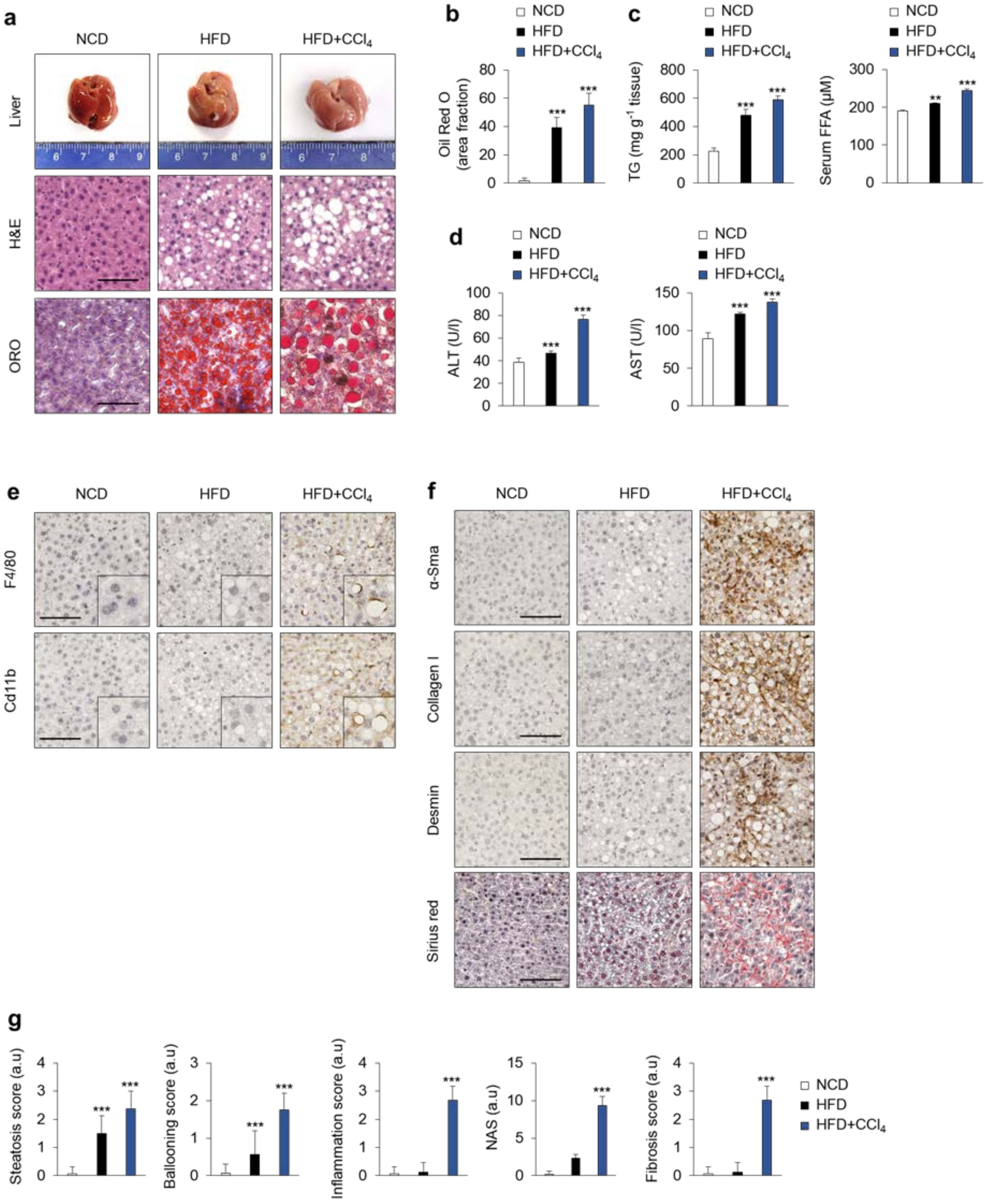

Supplementary Fig. 1. HFD+CCl4 fed mice developed NASH phenotype including hepatic steatosis, inflammation, and fibrosis. a-g C56BL/6 mice fed NCD, HFD alone or HFD+CCl4 (n = 10 group). **a** Representative histological images of H&E and

ORO staining. Scale bars, 100 µm. **b** Quantification of ORO positive area. **c**, **d** Serum concentration of TGs in the liver and serum FFA (**c**), ALT and AST (**d**). **e** Representative IHC images stained for F4/80 and Cb11b. High magnification of hCLS. Scale bars, 100 µm. **f** Representative IHC images of α-Sma, Collagen I, Desmin, and Sirius red staining. Scale bars, 100 µm. **g** The score for steatosis, ballooning, inflammation, NAFLD, and fibrosis. *$P < 0.05$, **$P < 0.01$, ***$P < 0.001$ determined by Student's *t*-test. Data represent mean ± SD.

Supplementary Fig. 2

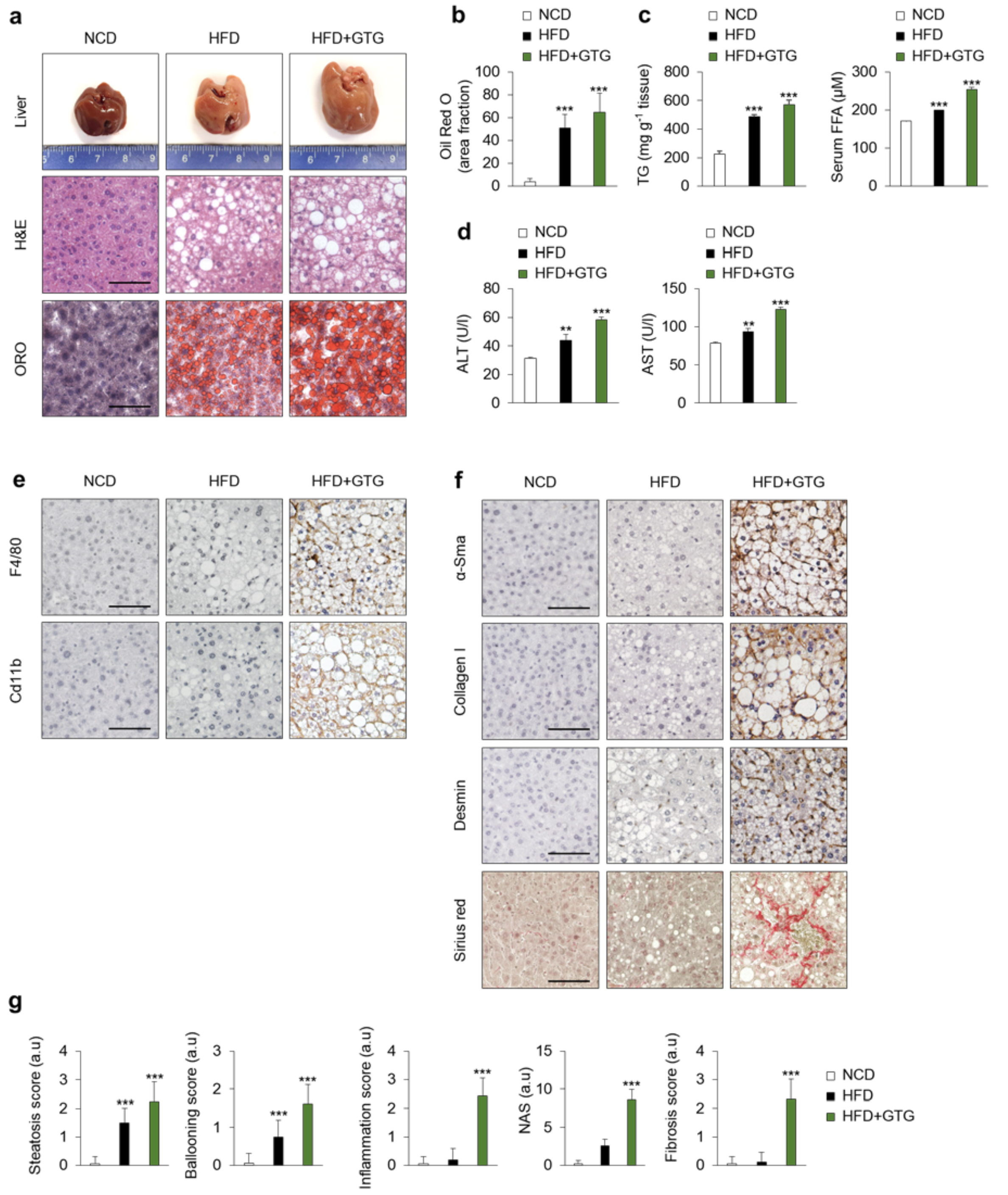

Supplementary Fig. 2. HFD+GTG fed mice developed NASH phenotype including hepatic steatosis, inflammation, and fibrosis. a-g C56BL/6 mice fed NCD, HFD alone or HFD+GTG (n = 9 group). **a** Representative histological images of H&E and

ORO staining. Scale bars, 100 µm. **b** Quantification of ORO positive area. **c**, **d** Serum concentration of TG in the liver and serum FFA (**c**), ALT and AST (**d**). **e** Representative IHC images stained for F4/80 and Cb11b. High magnification of hCLS. Scale bars, 100 µm. **f** Representative IHC images of α-Sma, Collagen I, Desmin, and Sirius red staining. Scale bars, 100 µm. **g** The score for steatosis, ballooning, inflammation, NAFLD, and fibrosis. **P* < 0.05, ***P* < 0.01, ****P* < 0.001 determined by Student's *t*-test. Data represent mean ± SD.

Supplementary Fig. 3

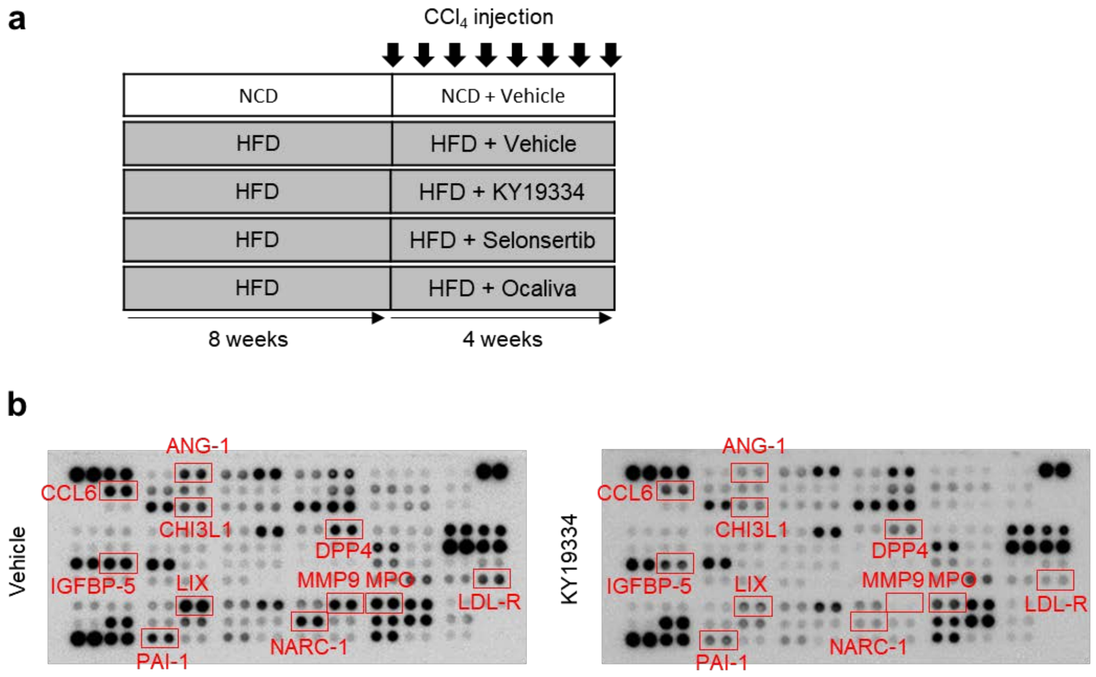

Supplementary Fig. 3. Experimental protocol and the evaluation of the metabolic effect of KY19334 on NASH mice model. a C57BL/6 mice fed NCD or HFD for 8 weeks and injected CCl_4 or vehicle (corn oil) were orally administered with KY19334, selonsertib, or ocaliva concentration at 25 mg/kg/d for 4 weeks (n = 10 per group). **b** The mouse cytokine and chemokine protein array in serum. ***$P < 0.001$ determined by Student's *t*-test. Data represent mean ± SD.

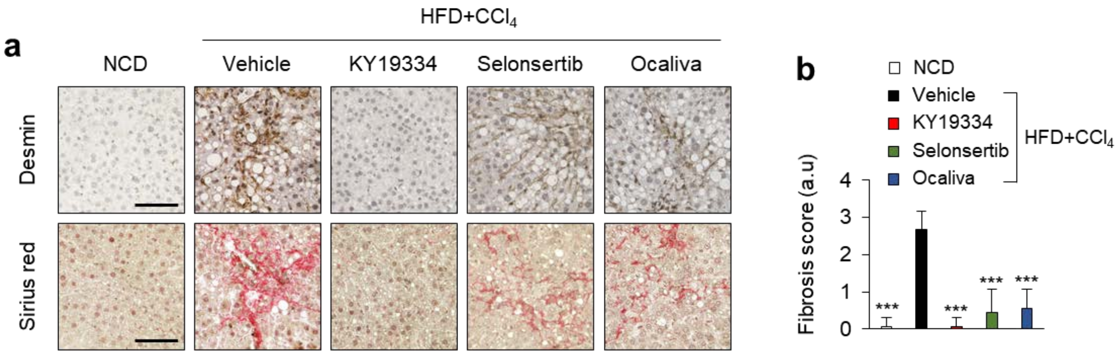

Supplementary Fig. 4. KY19334 treatment reduces and oxidative stress and cell death in the NASH mice. a-b C57BL/6 mice fed NCD or HFD+CCl_4 were orally administered with KY19334, selonsertib or ocaliva at 25 mg/kg/d for 4 weeks (n = 10 per group). **a** Representative IHC images of Desmin, and Sirius red staining. Scale bars, 100 µm. **b** Fibrosis score evaluated by Sirius red staining area. **P* < 0.05, ***P* < 0.01, ****P* < 0.001 determined by Student's *t*-test. Data represent mean ± SD.

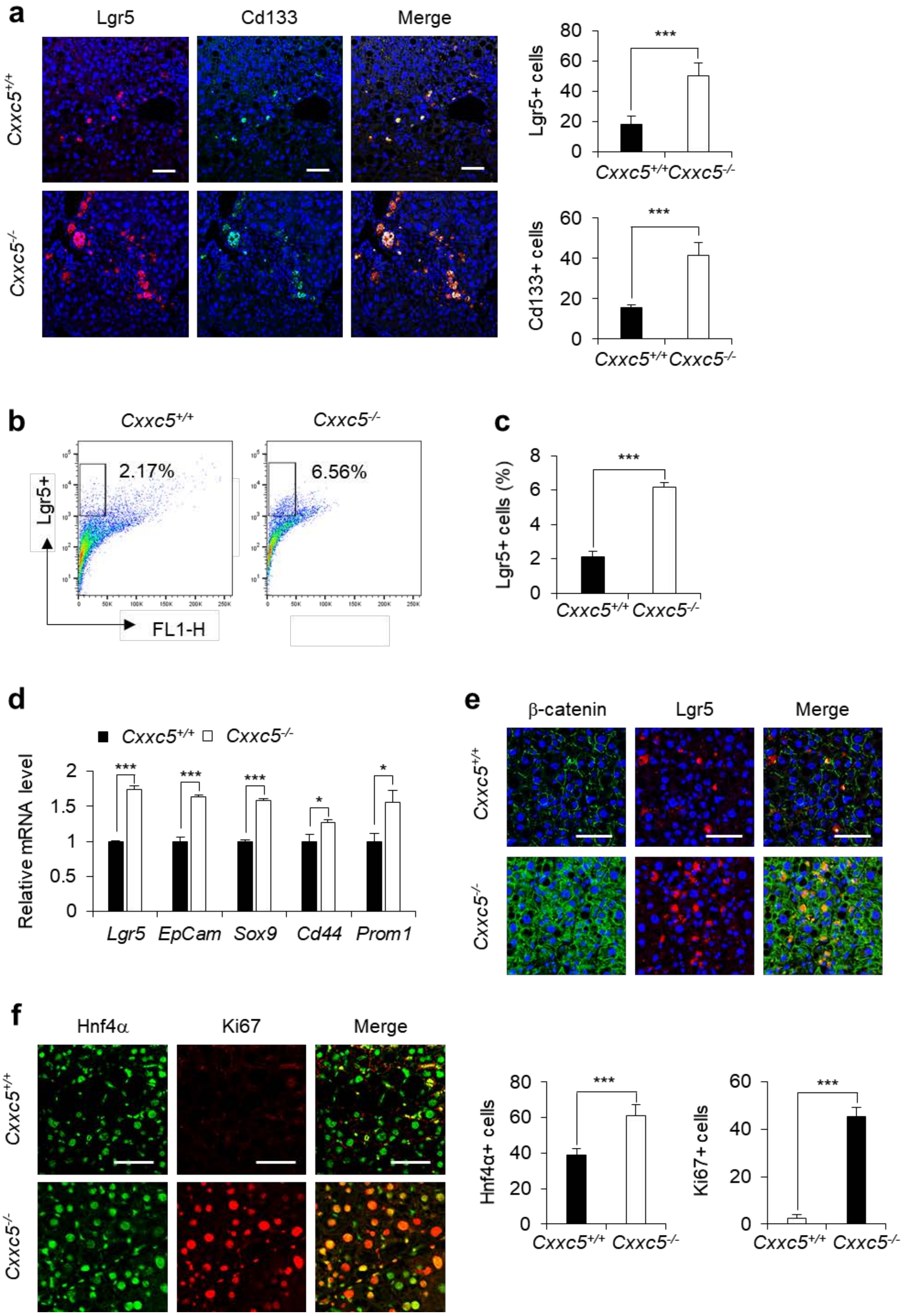
a
Lgr5
Cd133
Merge
Cxxc5+/+
Cxxc5-/-
Lgr5+ cells
80
60
40
20
0
***
Cd133+ cells
b
2.17%
6.56%
Lgr5+
FL1-H
c
Lgr5+ cells (%)
d
Relative mRNA level
Lgr5
EpCam
Sox9
Cd44
Prom1
e
β-catenin
f
Hnf4α
Ki67
Hnf4α+ cells
Ki67+ cells

Supplementary Fig. 5. Deletion of *Cxxc5* promotes liver regeneration in HFD+CCl_4-indudced NASH mice. a-f *Cxxc5*$^{+/+}$ and *Cxxc5*$^{-/-}$ mice were fed HFD or HFD+CCl_4 for 8 weeks for induction of NASH (n = 7 per group). The liver tissues were analyzed by various histochemical and biochemical methodologies. **a** Representative IHC images of Lgr5 and Cd133 (left panel). Quantification of Lgr5 and Cd133 cells (right panel). Scale bars, 100 µm. **b** Flow cytometry analysis of the expression of Lgr5. **c** Quantification of Lgr5 positive cells by flow cytometry analyses. **d** Relative mRNA expression of Lgr5 and liver progenitor cell markers. **e** Representative IHC images of β-catenin or Lgr5. Scale bars, 100 µm. **f** Representative IHC images of Hnf4α and Ki67 (left panel). Quantification of Hnf4α+ and Ki67+ cells (right panel). Scale bars, 100 µm. $^*P < 0.05$, $^{**}P < 0.01$, $^{***}P < 0.001$ determined by Student's *t*-test. Data represent mean ± SD.

Supplementary Fig. 6

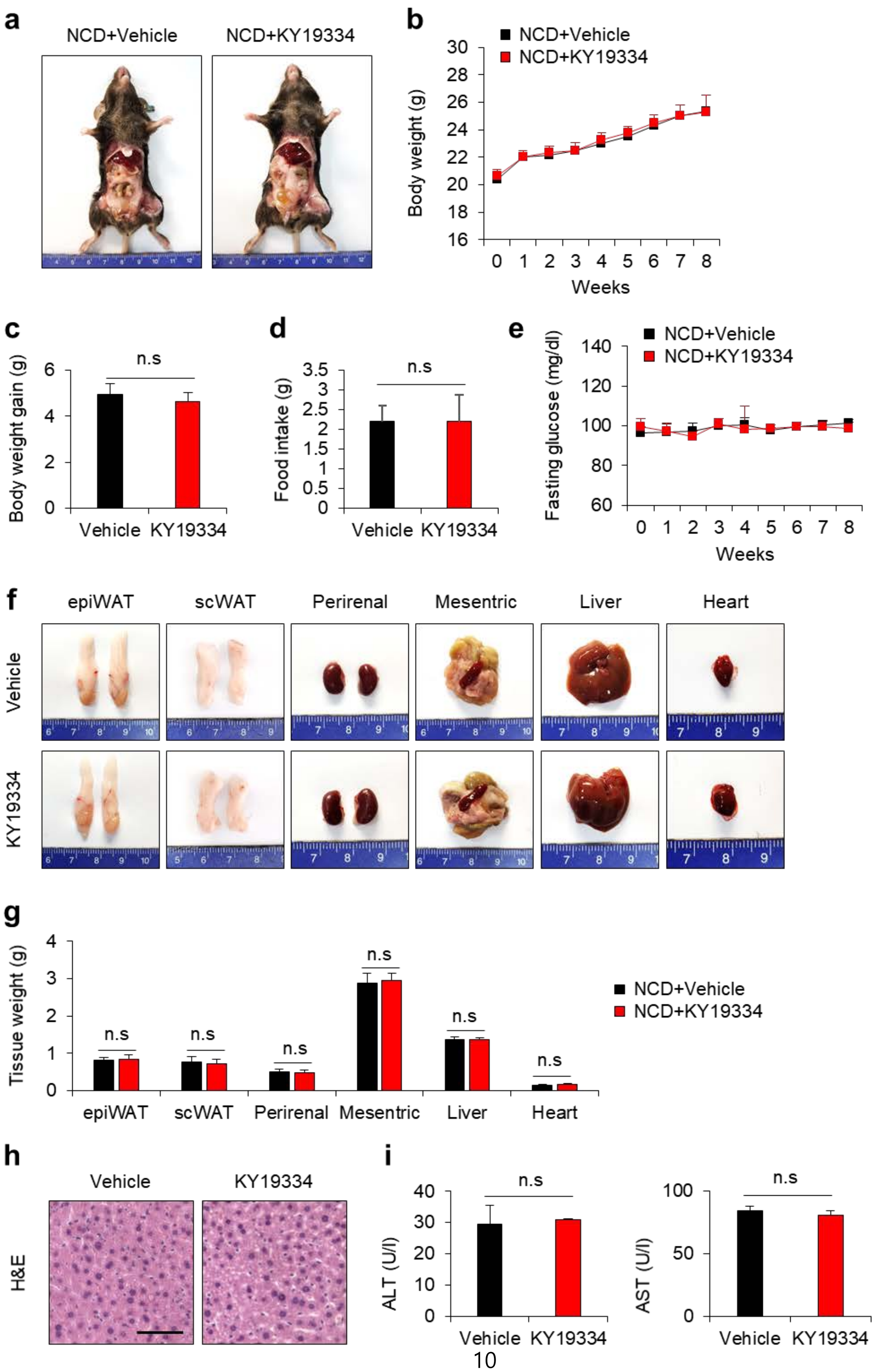

Supplementary Fig. 6. KY19334 treatment has no effect in NCD mice. a-i C57BL/6 mice fed NCD were orally administered KY19334 (25 mg/kg/d) for 8 weeks (n = 5 per group). **a** Representative photographs of vehicle- or KY19334-treated mice. **b** Body weight changes. **c** Body weight gain. **d** Food intake. **e** Fasting glucose. **f** Representative photographs of fat pads (epiWAT, scWAT), perirenal, mesenteric, liver and heart. **g** Wet weight of epiWAT, scWAT, perirenal, mesenteric, liver and heart. **h** Representative histological images of H&E staining. Scale bar, 100 µm. **i** Plasma concentration of ALT and AST. Data represent mean ± SD. n.s, not significant; determined using the Student's *t*-test.

Supplementary Table 1. Patient clinical and biochemical characteristics, related to Fig. 1a, b.

	Normal	NASH
Age	52.3 ± 18.58	45.4 ± 8.96
BMI (kg/m^2)	24.2 ± 3.37	44.4 ± 12.78
Leptin (mg/dl)	7.2 ± 6.86	31.1 ± 17.55
Adiponectin (mg/dl)	10.9 ± 5.78	6.9 ± 3.37
Fibrosis (0:1:2:3)	9:1:0:2	2:9:0:3
Inflammation (0:1:2:3)	11:1:0:0	0:8:5:1

Supplementary Table 2. Patient and biochemical information, related to Fig. 1c.

	Normal	NASH
Age	47.0 ± 13.29	46.7 ± 15.45
Macro Fat (%)	0 ± 0	52.9 ± 20.17
BMI	26.9 ± 3.11	41.2 ± 8.79

Supplementary Table 3. Sequences of the real-time PCR primers used in the study.

Gene	Forward	Reverse
Cxxc5	5'-CAAGAAGAAGCGGAAACGCTGC-3'	5'-TCTCCAGAGCAGCGGAAGGCTT-3'
Tcf7l2	5'-TGTGTACCCAATCACGACAGGAG-3'	5'-GATTCCGGTCGTGTGCAGAG-3'
Glp-1	5'-CCTGGTTGGTATCCCGGGA-3'	5'-CCGCTTCAGCTGAAGTCGCA-3'
Axin2	5'-TGGAGAGTGAGCGGCAGAGC-3'	5'-TGGAGACGAGCGGGCAGA-3'
Fosl1	5'-AACCGGAGGAAGGAACTGAC-3'	5'-CTGCAGCCCAGATTTCTCA-3'
Wisp1	5'-ATCGCCCGAGGTACGCAATAGG-3'	5'-CAGCCCACCGTGCCATCAATG-3'
Pparγ	5'-TGTGGGGATAAAGCATCAGGC-3'	5'-CCGGCAGTTAAGATCACACCTAT-3'
Cecpα	5'-GGTGGACAAGAACAGCAACGA-3'	5'-TGTCCAGTTCACGGCTCAGCT-3'
Srebp1	5'-GGAGCCATGGATTGCACATT-3'	5'-GGCCCGGGAAGTCACTGT-3'
Fas	5'-GCGATGAAGAGCATGGTTTAG-3'	5'-GGCTCAAGGGTTCCATGTT-3'
Scd-1	5'-CTGTACGGGATCATACTGGTTC-3'	5'-GCCGTGCCTTGTAAGTTCTG-3'
Acc	5'-CCTCCGTCAGCTCAGATACA-3'	5'-TTTACTAGGTGCAAGCCAGACA-3'
Cd36	5'-ATGGGCTGTGATCGGAACTG-3'	5'-GTCTTCCCAATAAGCATGTCTCC-3'
Tnfα	5'-CGGAGTCCGGGCAGGT-3'	5'-GCTGGGTAGAGAATGGATCA-3'
Mcp1	5'-ACTGAAGCCAGCTCTCTCTTCCTC-3'	5'-TTCCTTCTTGGGGTCAGCACAGAC-3'
Ifnγ	5'-TCAAGTGGCATAGATGTGGAAGAA-3'	5'-TGGCTCTGCAGGATTTTCATG-3'
F4/80	5'-CTTTGGCTATGGGCTTCCAGTC-3'	5'-GCAAGGAGGACAGAGTTTATCGTG-3'
Bax	5'-CGGCGAATTGGAGATGAACTG-3'	5'-GCAAAGTAGAAGAGGGCAACC-3'
Bcl-2	5'-AGCTGCACCTGACGCCCTT-3'	5'-GTTCAGGTACTCAGTCATCCAC-3'
Bcl-x	5'-AGGTTCCTAAGCTTCGCAATTC-3'	5'-TGTTTAGCGATTCTCTTCCAGG-3'
P21	5'-AGATCCACAGCGATATCCAGAC-3'	5'-ACCGAAGAGACAACGGCACACT-3'
α*-Sma*	5'-GTCCCAGACATCAGGGAGTAA-3'	5'-TCGGATACTTCAGCGTCAGGA-3'
Col1a1	5'-AAGGTATTGCTGGACAGCGT-3'	5'-TGTTTGCCAGGTTCACCAGA-3'
Mmp3	5'-ATTCCATGGAGCCAGGCTTT-3'	5'-CATTTGGGTCAAACTCCAAC-3'
Tgfβ	5'-TGACGTCACTGGAGTTGTACGG-3'	5'-GGTTCATGTCATGGATGGTGC-3'
Sirt1	5'-TTGGCACCGATCCTCGAAC-3'	5'-CCCAGCTCCAGTCAGAACTAT-3'

Pgc-1α	5’-AGCCGTGACCACTGACAACGAG-3’	5’-GCTGCATGGTTCTGAGTGCTAAG-3’
Pparδ	5’-TCCATCGTCAACAAAGACGGG-3’	5’-ACTTGGGCTCAATGATGTCAC-3’
Ucp1	5’-AGGCTTCCAGTACCATTAGGT-3’	5’-CTGAGTGAGGCAAAGCTGATTT-3’
Prdm16	5’-CCACCAGCGAGGACTTCAC-3’	5’-GGAGGACTCTCGTAGCTCGAA-3’
Elovl3	5’-TTCTCACGCGGGTTAAAAATGG-3’	5’-GAGCAACAGATAGACGACCAC-3’
Cox8b	5’-GAACCATGAAGCCAACGACT-3’	5’-GCGAAGTTCACAGTGGTTCC-3’
Cd137	5’-CCTTGCAGGTCCTTACCTTGT-3’	5’-GTTGCTTGAATATGTGGGGGA-3’
Tmem26	5’-ATGGTGCATTTCAAGAAGCC-3’	5’-GCTCACCCTCAAGTTCAAGC-3’
Tbx1	5’-CTGTGGGACGAGTTCAATCAG-3’	5’-TTGTCATCTACGGGCACAAAG-3’
Lgr5	5’-GACAATGCTCTCACAGAC-3’	5’-GGAGTGGATTCTATTATTATGG-3’
EpCam	5’-CCTGAGAGTGAACGGAGAGC-3’	5’-GACACCACCACAATGACAGC-3’
Sox9	5’-CGACTACGCTGACCATCAGA-3’	5’-AGACTGGTTGTTCCCAGTGC-3’
Cd44	5’-TGAAACATGCAGGTATGGGT-3’	5’-GCTGAGGCATTGAAGCAATA-3’
Prom1	5’-TCATCGCTGTGGTCGTCATTG-3’	5’-GTCCGCTGGTGTAGTGTTGTAG-3’
